# Epigenetic dysregulation in various types of cells exposed to extremely low-frequency magnetic fields

**DOI:** 10.1007/s00441-021-03489-6

**Published:** 2021-07-21

**Authors:** Gianfranco Giorgi, Brunella Del Re

**Affiliations:** Department of Pharmacy and Biotechnology, Alma Mater Studiorum University of Bologna, via Selmi 3, 40126 Bologna, Italy

**Keywords:** Epigenetics, DNA methylation, Histone modifications, MicroRNA, Electromagnetic, magnetic field

## Abstract

Epigenetic mechanisms regulate gene expression, without changing the DNA sequence, and establish cell-type-specific temporal and spatial expression patterns. Alterations of epigenetic marks have been observed in several pathological conditions, including cancer and neurological disorders. Emerging evidence indicates that a variety of environmental factors may cause epigenetic alterations and eventually influence disease risks. Humans are increasingly exposed to extremely low-frequency magnetic fields (ELF-MFs), which in 2002 were classified as possible carcinogens by the International Agency for Research on Cancer. This review summarizes the current knowledge of the link between the exposure to ELF-MFs and epigenetic alterations in various cell types. In spite of the limited number of publications, available evidence indicates that ELF-MF exposure can be associated with epigenetic changes, including DNA methylation, modifications of histones and microRNA expression. Further research is needed to investigate the molecular mechanisms underlying the observed phenomena.

## Introduction

In recent years, an important new research area has emerged, dealing with the interplay between environment and molecular epigenetic mechanisms and the possible impact on human health (Cavalli and Heard 2019; Perera et al. 2020).

Epigenetic mechanisms include DNA methylation (Greenberg and Bourc’his 2019), post-translational modification of tail domains of histone proteins (Jenuwein and Allis 2001) and noncoding RNAs (ncRNAs) (Holoch and Moazed 2015). DNA methylation is a process of adding a methyl group to DNA, which in mammalian cells occurs mainly at the C5 position of cytosine (5meC) within the CpG dinucleotide (Cooper and Krawczak 1989). Histones are nuclear proteins that package DNA in nucleosomes, the units of the chromatin structure. Their N-terminal long tail can be subjected to a variety of post-translational modification reactions, including acetylation, methylation, phosphorylation, citrullination, sumoylation, glycosylation, ADP-ribosylation, ubiquitination, biotinylation, crotonylation and deamination (Sadakierska-Chudy and Małgorzata 2015). DNA methylation and histone modification are governed by effector proteins named writers, readers and erasers, which respectively add, bind or remove chemical groups (Allis and Jenuwein 2016). ncRNAs are functional RNA molecules that are transcribed from DNA, but not translated into proteins. They belong to several classes, which differ in structure and function, including microRNAs (miRNAs, miRs), short interfering RNAs (siRNAs), PIWI-interacting RNAs (piRNAs) and long noncoding RNAs (lncRNAs). Effects of environmental agents on ncRNA levels are generally investigated by evaluating miR expression. miRs are single-stranded RNAs of approximately 21–23 nucleotides and are involved in gene silencing within the RNA interference pathway. They are partially complementary to one or more messenger RNA (mRNA) molecules; each molecule can modulate the expression of many pathways interacting with many mRNAs causing translational inhibition or mRNA destabilization (Pillai et al. 2007; Wang et al. 2013).

DNA methylation, chemical modification of histone proteins and noncoding RNAs work cooperatively through the recruitment of transcriptional repressor/activator proteins (Voon and Gibbons 2016). They establish patterns of modifications associated with open or closed chromatin structures where DNA is respectively accessible or not accessible to the transcriptional machinery (Vaissière et al. 2008; Rose and Klose 2014; Hanly et al. 2018). These patterns determine the epigenetic state of the genome, named epigenome, which varies by cell type and over time as a consequence of the developmental process (Reik et al. 2001; Bintu et al. 2016). Epigenetic marks are usually heritable, allowing for maintenance of cell identity, but can also be reversible, allowing for developmental plasticity (Lee et al. 2014).

Accumulated evidence has shown an association between epigenetic alterations and pathological conditions, including cancer (Lehmann 2014; Herceg et al. 2018; Perdigoto 2019) and neurodegenerative diseases (Schroeder et al. 2011; Hwang et al. 2017), as well as between epigenetic alterations and ageing (Sen et al. 2016; Horvath and Raj 2018). It is unclear what triggers these epigenetic dysregulations; however, emerging evidence indicates that a variety of environmental factors may have an epigenetic impact (Cortessis et al. 2012). Large amounts of data were collected on this topic, suggesting that epigenetic alterations could represent a pathway by which environmental factors influence disease risks and ageing. In particular, early embryonic development is highly vulnerable to epigenetic changes caused by environmental conditions. At this stage, global changes in the epigenetic landscape occur and drive cell-fate decisions: stem cells differentiate into different cell lineages, acquiring cell-type-specific epigenetic signatures which are responsible for differential gene expression and specific cell functions. During this process, environmental stressors can affect epigenetic patterns, leading, later in life, to adverse health effects, according to the theory known as developmental origin of health and disease (DOHaD) (Barouki et al. 2012, 2018).

Many works analysed alterations of epigenetic marks in human cells exposed to various environmental agents. Several reviews dealing with this topic have been published in the last 10 years (Bollati and Baccarelli 2010; Alegría-Torres et al. 2011; Collotta et al 2013; Lin et al. 2016; Cui et al. 2017; Alfano et al. 2018; Mahna et al. 2018; Martin and Fry 2018; Pan et al. 2018; Ferrari et al. 2019; Rider and Carlsten 2019; Cheng et al. 2020; Chung and Herceg 2020). However, none of them focused on the epigenetic effects of the exposure to extremely low-frequency magnetic fields (ELF-MFs), which are today an ubiquitous environmental factor.

Humans are increasingly exposed to ELF-MFs generated by everyday electrical devices and powerlines; therefore, concerns about potential health risks have been increasing in recent decades. In 2002, ELF-MFs have been classified as possible carcinogens (2B) for humans by the International Agency for Research on Cancer (IARC) on the basis of epidemiological studies that associated ELF-MF exposure with an increased risk for childhood leukaemia (IARC 2002). Since then, various epidemiological and experimental studies have been performed to evaluate the carcinogenicity of ELF-MF exposure (Juutilainen et al. 2006; Erdal et al. 2007; Magnani et al. 2014; Salvan et al. 2015; Schüz et al. 2016; Soffritti et al. 2016a, b; Campos-Sanchez et al. 2019; Carles et al. 2020), but the results were not conclusive and the question is still unanswered. Recently ELF-MF exposure has been associated with an increased risk of neurological disorders (Qiu et al. 2004; Consales et al. 2012; Brouwer et al. 2015; Pedersen et al. 2017; Jalilian et al. 2018; Huss et al. 2018), but the underlying molecular mechanisms are not fully understood.

Several studies showed that electromagnetic fields can modulate processes that involve epigenetic mechanisms, such as cell commitment (Maziarz et al. 2016) and neuronal and osteogenic differentiation (Leone et al. 2015). Because of these effects, magnetic fields are considered of interest for therapeutic interventions, including repair of tissue injury and development of bone (Varani et al. 2021).

Therefore, the study of the impact of the ELF-MF exposure on epigenetic marks could be useful for both the above aspects: public health protection and therapeutic use.

The purpose of the present review is to summarize the results arising from studies that investigated the link between the exposure to ELF-MF and epigenetic alterations and to explore possible future developments.

## ELF-MF exposure effects on epigenetic marks

Only a modest number of studies have been performed up to now to assess effects of ELF-MF exposure on epigenetics. They are very heterogeneous in experimental designs and exposure systems, as summarized in Table 1. These studies are here grouped on the basis of the cell type/tissue/organ analysed and the research question.

**Table 1:** Effect of the exposure to ELF-MF on epigenetic marks. Models: *A549* human adenocarcinomic alveolar basal epithelial cell line, *BE(2)C* human neuroblastoma cell line, *BMSCs* human bone marrow mesenchymal stem cells, *GC-2* mouse spermatocyte-derived cells, *iPSC* induced pluripotent stem cells, *Jurkat* human acute T cell leukaemia cell line, *LLC* mouse Lewis lung cancer cells, *OPCs* oligodendrocyte precursor cells (obtained from the cerebral cortex of rat pups), *NSCs* neural stem cells (isolated from the hippocampi of newborn mice), *PBMCs* peripheral blood mononuclear cells, *PCNs* mouse primary cortical neurons, *SH-SY5Y* human neuroblastoma cell line, *T98G* human glioblastoma cell line

Investigation on	Model	Exposure details	QE*			Epigenetic marks			Ref.
			a	b	c	DNA methylation	miRNAs (for details see Table 2)	Histone marks	
Spermatocyte-derived cells	GC-2	50 Hz, 1–3 mT (SiMF) 5 min on/10 min off, for 72 h	+	+	−	Global methylation decreased at 1 mT and increased at 2 and 3 mT (by ELISA-based colorimetric quantitative assay) 296 differentially methylated sites at 1 mT and 70 at 3 mT (by DNA methylation chip analysis)	ND	ND	Liu et al. (2015b)
						ND	Up- and downregulation depending on magnetic flux density (entries 2, 11, 12, 21, 22, 23, 24, 26, 27, 28, 31–45 in Table 2)	ND	Liu et al. (2015a)
						No effect on the methylation of the host gene of miR-26b-5p (CTDSP1) (by MSP)	Up- and downregulation depending on magnetic flux density (entry 7 in Table 2)	ND	Liu et al. (2016)
Haematopoietic cells	- Jurkat - CD34+ haematopoietic stem cells	50 Hz, 1 mT (SiMF) 5 min on/10 min off for 72 h (Jurkat); for 5 days (CD34)	+	+	+	No effect on the methylation patterns of CD34+ haematopoietic stem cells (by DNA bisulphite conversion and methylation arrays)	ND	No effect on H3K4me2 H3K27me3 (by ChIP-seq)	Manser et al. (2017)
Lung cells	- LLC cells - A549 cells - Mice inoculated with LLC	7.5 Hz, 0.4 T (RMF) for 6 days (LLC); 2 h a day, for 35 days (mice)	+	−	−	ND	Upregulation (entry 13 in Table 2)	ND	Ren et al. (2017)
Brain cells	NSCs	50 Hz, 1 mT (SiMF) for 24 h	+	−	−	ND	ND	Increased acetylation of H3K9 (by ChIP assay)	Leone et al. (2014)
	T98G +/− TMZ	75 Hz, 2 mT (PEMF) for 1 h	+	−	−	ND	Up- and downregulation (entries 3, 4, 30 in Table 2)		Pasi et al. (2016)
	PBMCs, from 13 AD patients	75 Hz, 3 mT (PEMF) for 15, 30, 60 min	+	−	−	ND	Downregulation (entries 8, 18, 29 in Table 2)	ND	Capelli et al. (2017)
	BE(2)C	50 Hz, 1 mT (PEMF) for 24, 48 h +/− OS (300 μM H_2_O_2_)	+	+	+	Transient decrease and increase of the methylation of several CpGs located in L1 5′UTR, mainly under ELF + OS (by MassARRAY EpiTYPER technology, Sequenom)	ND	ND	Giorgi et al. (2017)
	- SH-SY5Y - PCNs	50 Hz, 1 mT (SiMF) for 4–72 h	+	+	+	Increase of the methylation of CpGs within miR-34b/c promoter (by DNA bisulphite conversion and pyrosequencing of the 7 CpGs of a fragment within the miR-34b/c promoter)	Downregulation (entries 14,15 in Table 2)	ND	Consales et al. (2018)
	Brain cells from male/female rats young (3 weeks old) and mature (10 weeks old)	50 Hz, 1 mT (SiMF) 4 h/day for 60 days	+	−	−	ND	Up- and Downregulation (entries 1, 6, 9, 16, 17, 19 in Table 2)	ND	Erdal et al. (2018)
	SH-SY5Y - Proliferating +/− MPP - Differentiated (dopaminergic) +/− MPP	50 Hz, 1 mT (SiMF) up to 72 h	+	+	+	No effect on the methylation of 5 CpGs of L1, 4 CpGs of SATα, 5 CpGs of ALU (by DNA bisulphite conversion and pyrosequencing analysis)	ND	ND	Benassi et al. (2019)
	OPCs	50 Hz, 1.8 mT (PEMF) for 2 h twice a day, for 3, 7, 14, 21 days	+	+	−	ND	Upregulation (entry 25 in Table 2)	ND	Yao et al. (2019)
iPSC generation	iPSCs from mouse tail tip fibroblasts	50 Hz, 1 mT (SiMF) for up to 30–45 days	+	−	−	ND	ND	Increased H3K4me3 (by ChIP assay)	Baek et al. (2014)
Osteogenic differentiation	BMSCs	75 Hz, 1.5 mT (PEMF) for 21 days	+	−	−	ND	Up- and downregulation (entries 5, 10, 20 in Table 2)	ND	De Mattei et al. (2020)

QE*: For each study, the presence (+) or the absence (−) of three “quality criteria” of the exposure (QE), chosen on the basis of general guidelines (Kuster and Schönborn, 2000) is reported: *a*, appropriate description of the ELF-MF homogeneity, declaring the variation of physical parameters during the exposure; *b*, appropriate sham control, able to take into account the microenvironment in the exposure device (i.e. coil systems using separated strand cables wrapped in parallel to enable the currents to flow also in antiparallel directions); *c*, blind conditions, allowing the treatment group to be unknown until after data analysis in order to avoid biasing in results

*AD* Alzheimer disease, *ELISA* enzyme-linked immunosorbent assay, *ChIP* chromatin immunoprecipitation, *MPP* 1-methyl-4 phenylpyridinium, *MSP* methylation-specific PCR, *ND* not done, *OS* oxidative stress, *PEMF* pulsed magnetic field, *RMF* rotating magnetic field, *SiMF* sinusoidal magnetic field, *TMZ* temozolomide

### Investigations on spermatocyte-derived cells

Some evidence suggested that ELF-MFs could affect semen quality in animals and humans, causing dysfunction of the male reproductive system (Iorio et al. 2007; Kim et al. 2009; Roychoudhury et al. 2009). The underlying possible molecular mechanisms remain unknown. The research question of the three studies reported below, conducted by the same team, was to find out if epigenetic perturbations could play a role in these phenomena.

In the first study, Liu et al. (2015b) assessed whether ELF-MF exposure could affect the levels of DNA methylation in spermatocyte cells. To this purpose, starved mouse spermatocyte-derived GC-2 cells were subjected to an exposure of 50 Hz ELF-MF at various magnetic flux densities for 72 h. Starvation was induced by culturing the cells in serum-free medium for 12 h before the exposure. The following endpoints were analysed: (1) the levels of global DNA methylation; (2) the levels of mRNAs and proteins of DNMT1, DNMT3a and DNMT3b methyltransferases, which are writers of DNA methylation patterns; (3) screening of differential methylated sites in detail. The evaluation of the global DNA methylation showed that ELF-MF-exposed GC-2 cells acquired aberrant methylation levels, depending on the magnetic flux density used: a decrease at 1 mT and an increase at 2 mT and 3 mT, as compared with the sham-exposed controls, were observed. Coherently, mRNA and protein expressions of DNMT1 and DNMT3b decreased at 1 mT and increased at 3 mT. Differently, no influence on the expression of DNMT3a was observed. The result was strengthened by DNA methylation chip analysis that showed a number of differentially methylated sites, both hypermethylated and hypomethylated, in the 1-mT and 3-mT-exposed samples as compared with the control group. Gene expression was also evaluated by microarrays and then confirmed and validated by using RT-qPCR: a total of 84 differentially expressed genes in 1-mT-exposed samples and 324 differentially expressed genes in 3-mT-exposed samples were observed as compared with the control group.

In the second study (Liu et al. 2015a), the authors used the same model and experimental conditions to investigate the possible effect of ELF-MFs on miR expression, which is known to be involved in spermatogenesis and male fertility (Suh and Blelloch 2011). After exposure, miR levels were evaluated using microarray technology. To validate the miR array data, several differentially expressed miRs were selected for RT-qPCR quantification. Those miRs whose expression significantly changed, compared with the sham group, are reported in Table 2. The authors applied a network analysis to predict putative miR target genes and their biological functions and found that many of the predicted miR target genes were involved in critical cellular pathways. In particular, they observed that miR-494-3p was the most highly upregulated miR (+2.3 times at 1 mT; +3.3 times at 3 mT) among the differentially expressed miRs, and it may act as an oncogene by targeting genes related to the cell cycle and apoptosis (Ohdaira et al. 2012). In several cases, the effect depended on the magnetic flux density (Table 2).

**Table 2:** List of miRs found as affected by ELF-MF exposure. Model: *A549* adenocarcinomic human alveolar basal epithelial, *BMSCs* human bone marrow mesenchymal stem cells, *GC-2* mouse spermatocyte-derived cells, *LLC* mouse Lewis lung cancer cells, *OPCs* oligodendrocyte precursor cells (obtained from the cerebral cortex of rat pups), *PBMCs* peripheral blood mononuclear cells, *PCNs* mouse primary cortical neurons, *SH-SY5Y* human neuroblastoma cell line, *T98G* human glioblastoma cell line

		Effect	Model and exposure	Ref.
1	miR-9-5p	- Downregulation* in blood cells from young female/male rats - Upregulation* in brain cells from young female and mature male rats	Male/female young/mature rats exposed to SiMF of 50 Hz, 1 mT (4 h/day for 60 days)	Erdal et al. (2018)
2	miR-16-1-3p	- Downregulation* at 3 mT	GC-2 exposed to SiMF of 50 Hz, 1 mT, 2 mT, 3 mT (5 min on/10 min off, for 72 h)	Liu et al. (2015a)
3	miR-17-3p	- Downregulation* in cells subjected to combined PEMF + TMZ treatment	T98G cells +/− TMZ, exposed to PEMF of 75 Hz, 2 mT for 1 h	Pasi et al. (2016)
4	miR-21-3p	- Downregulation* in cells subjected to combined PEMF + TMZ treatment	T98G cells +/− TMZ, exposed to PEMF of 75 Hz, 2 mT for 1 h	Pasi et al. (2016)
5	miR-26a	- Upregulation*	BMSCs exposed to PEMF of 75 Hz, 1.5 mT for 21 days	De Mattei et al. (2020)
6	miR-26b-5p	- Downregulation* in blood and brain cells from young female rats	Male/female young/ mature rats exposed to SiMF of 50 Hz, 1 mT (4 h/day for 60 days)	Erdal et al. (2018)
7	miR-26b-5p	- Upregulation* at 2 mT - Downregulation* at 3 mT	GC-2 exposed to SiMF of 50 Hz, 1 mT, 2 mT, 3 mT (5 min on/10 min off, for 72 h)	Liu et al. (2016)
8	miR-26b-5p	- Downregulation* (progressive reduction with the increasing time of exposure; differences between untreated and treated cells were not statistically significant)	PBMC from 13 AD patients, exposed to PEMF of 75 Hz, 3 mT for 15, 30, and 60 min	Capelli et al. (2017)
9	miR-29a-3p	- Downregulation* in blood and brain cells from young female rats, in blood cells from young male rats, in brain cells from mature male rats	Male/female young/ mature rats exposed to SiMF of 50 Hz, 1 mT (4 h/day for 60 days)	Erdal et al. (2018)
10	miR-29b	- Upregulation*	BMSCs exposed to PEMF of 75 Hz, 1.5 mT for 21 days	De Mattei et al. (2020)
11	miR-29b-2-5p	- Upregulation at 1 mT	GC-2 exposed to SiMF of 50 Hz, 1 mT, 2 mT, 3 mT (5 min on/10 min off, for 72 h)	Liu et al. (2015a)
12	miR-30e-5p	- Upregulation* at 1 mT - Downregulation*at 3 mT		
13	miR-34a	- Upregulation* in cell lines and in tumour tissue of LLC mouse	- LLC and A549, exposed to RMF of 7.5 Hz, 0.4 T (2 h a day for 6 days) - Mice inoculated with LLC, exposed to RMF of 7.5 Hz, 0.4 T (2 h a day for 35 days)	Ren et al. (2017)
14	miR-34b	- Downregulation* in SH-SY5Y cells and in PCN cells	SH-SY5Y and PCNs exposed to SiMF of 50 Hz, 1 mT (for 4–72 h)	Consales et al. (2018)
15	miR-34c	- Downregulation* in SH-SY5Y cells and in PCN cells		
16	miR-106b-5p	- Downregulation* in blood and brain cells from young female rats - Upregulation* in brain cells from young male rats	Male/female young/ mature rats exposed to SiMF of 50 Hz, 1 mT (4 h/day for 60 days)	Erdal et al. (2018)
17	miR-107	- Downregulation* in blood and brain cells from young female rats - Upregulation* in brain cells from young male rats and in blood cells from mature male rats		
18	miR-107	- Downregulation* (progressive reduction with the increasing time of exposure; differences between untreated and treated cells were not statistically significant)	PBMC from 13 AD patients, exposed to PEMF of 75 Hz, 3 mT for 15, 30, and 60 min	Capelli et al. (2017)
19	miR-125a-3p	- Downregulation* in blood and brain cells from young female rats - Upregulation* in brain cells from young male rats and in blood cells from mature male rats	Male/female young/ mature rats exposed to SiMF of 50 Hz, 1 mT (4 h/day for 60 days)	Erdal et al. (2018)
20	miR-125b	- Downregulation*	BMSCs exposed to PEMF of 75 Hz, 1.5 mT for 21 days	De Mattei et al. (2020)
21	miR- 122-5p	- Downregulation at 1 mT	GC-2 exposed to SiMF of 50 Hz, 1 mT, 2 mT, 3 mT (5 min on/10 min off, for 72 h)	Liu et al. (2015a)
22	miR-196b-5p	- Upregulation* at 1 mT - Downregulation* at 2 mT and 3 mT		
23	miR-210-5p	- Upregulation* at 1 mT - Downregulation* at 2 mT and 3 mT		
24	miR-211-3p	- Upregulation at 1 mT and at 3 mT		
25	miR-219-5p	- Upregulation*	OPCs exposed to PEMF of 50 Hz and 1.8 mT for 2 h twice a day for 3, 7, 14, 21 days	Yao et al. (2019)
26	miR-224-5p	- Upregulation* at 1 mT - Downregulation* at 2 mT and 3 mT	GC-2 exposed to 50 Hz, 1 mT, 2 mT, 3 mT (5 min on/10 min off, for 72 h)	Liu et al. (2015a)
27	miR-322-5p	- Downregulation* at 3 mT		
28	miR-335-5p	- Upregulation at 3 mT		
29	miR-335-5p	- Downregulation* (progressive reduction with the increasing time of exposure; differences between untreated and treated cells were not statistically significant)	PBMC from 13 AD patients, exposed to PEMF of 75 Hz, 3 mT for 15, 30, and 60 min	Capelli et al. (2017)
30	miR-421-5p	- Upregulation* in PEMF-exposed cells and in TMZ-treated cells - Downregulation* in cells subjected to PEMF + TMZ	T98G cells +/− TMZ, exposed to PEMF of 75 Hz, 2 mT for 48 h	Pasi et al. (2016)
31	miR-455-3p	- Downregulation* at 1 mT and 3 mT - Upregulation* at 2 mT	GC-2 exposed to SiMF of 50 Hz, 1 mT, 2 mT, 3 mT (5 min on/10 min off, for 72 h)	Liu et al. (2015a)
32	miR-466i-5p	- Upregulation at 1 mT		
33	miR-467f	- Downregulation* at 3 mT		
34	miR-494-3p	- Upregulation at 1 mT and at 3 mT		
35	miR-504-3p	- Downregulation* at 3 mT - Upregulation* at 2 mT		
36	miR-665-5p	- Downregulation* at 3 mT		
37	miR-667-5p	- Upregulation at 3 mT		
38	miR-669c-5p	- Upregulation* at 1 mT - Downregulation* at 2 mT and 3 mT		
39	miR-1931	- Upregulation at 1 mT and at 3 mT		
40	miR-1955-3p	- Upregulation* at 1 mT		
41	miR-1965	- Downregulation* at 1 mT		
42	miR-3072-5p	- Upregulation at 3 mT		
43	miR-3084-3p	- Downregulation* at 3 mT		
44	miR-3092-3p	- Upregulation at 1 mT and at 3 mT		
45	miR-5128	- Upregulation at 3 mT		

*AD* Alzheimer disease, *PEMF* pulsed magnetic field, *RMF* rotating magnetic field, *SiMF* sinusoidal magnetic field, *TMZ* temozolomide

^*^Validated using real-time qPCR

In the third study (Liu et al. 2016), the investigation focused on miR-26b-5p, which showed increased expression at 2 mT, decreased expression at 3 mT, and no changes at 1 mT. Among the 85 putative target genes of miR-26b-5p, identified by computational prediction, an interesting binding site for miR-26b was found in the 5′ untranslated region (UTR) of cyclin D2 (CCND2) mRNA. CCND2 is a crucial cell cycle regulatory gene, and its aberrant expression has been reported in several cancer tissues and cell lines (Ando et al. 1993; Bartkova et al. 2001). The authors observed that the expression of CCND2 was negatively correlated with the expression of miR-26b-5p: indeed, CCND2 expression decreased at 2 mT, increased at 3 mT, remained unaltered at 1 mT. To investigate the potential mechanism of the deregulation of miR-26b-5p, DNA methylation of its host gene (CTDSP1) was evaluated, but no significant change was found indicating that it was not involved in the phenomenon.

The authors concluded that the regulation of miRs, and in particular of miR-26b-5p-CCND2-mediated cell cycle regulation, as well as the alterations of global methylation, related to DNMT altered expression, could play a role in the biological effects of intermittent 50 Hz ELF-MF exposure on spermatocyte-derived cells. However, no hypothesis was formulated about the different responses to different magnetic flux densities.

### Investigations on haematopoietic cells

Epidemiological studies have associated ELF-MF exposure with an increased risk for childhood leukaemia (IARC 2002; Schüz et al. 2016). The research question of the study carried out by Manser et al. (2017) was to explore if changes of epigenetic marks, such as histone modifications and DNA methylation, could play a role in the onset of the disease by driving haemopoietic cell dysfunction.

They used a human leukaemic cell line (Jurkat cells) and in vitro haematopoietic differentiating cells (CD34+ cord blood cells differentiating to neutrophilic lineage), which were exposed intermittently to ELF-MF (50 Hz, 1 mT). Before and after ELF-MF exposure, active (H3K4me2) and repressive (H3K27me3) histone modifications were analysed. Results indicated that the global patterns of H3K4me2 and H3K27me3 modifications in both Jurkat and haematopoietic differentiating cells was unaffected by ELF-MF exposure. Haematopoietic differentiating cells were subjected to genome-wide methylation analysis at single CpG sites. As expected, the pattern of DNA methylation changed dramatically during neutrophilic granulopoiesis, but no significant difference in methylation levels was observed between ELF-MF-exposed and control samples. So the conclusion was that ELF-MF exposure did not influence the formation of cell-type-specific DNA methylation patterns.

### Investigations on lung cells

Several studies have shown that specific electromagnetic field exposure can inhibit proliferation of cancer cells in vitro and cause decrease of tumour volume and longer survival time in vivo (Wang et al. 2011; Zhu et al. 2020). Ren et al. (2017) investigated whether changes of miR expression could play a role in these antitumour effects, carrying out both in vitro and in vivo experiments. In particular, they focused on miR-34a, since it is known to be downregulated in several cancer cells, including lung cancer (Xue et al. 2014; Cortez et al. 2016). They used a rotating magnetic field (RMF) of 7.5 Hz and 0.4 T. Differently from the other types of dynamic magnetic fields, the direction of the magnetic field in RMF is constantly changing. This specific type of exposure, which hardly occurs in a normal environment, has been used in various studies to explore therapeutic use of magnetic fields (Chen et al. 2016; Zhan et al. 2020).

The most interesting observation was that mice inoculated with Lewis lung cancer (LLC) cells and exposed to RMF for 35 days showed decreased tumour growth and increased levels of miR-34a in tumour tissue as compared with sham-exposed groups. An increase of miR-34a levels was found also in LLC cells and A549 cells cultured in vitro and subjected to RMF exposure for 2 and 4 days. A subsequent investigation of molecular mechanisms provided evidence that an iron-p53-miR-34a-E2F1/E2F3 pathway could be involved in the antitumour effect induced by electromagnetic exposure. The chain of events was as follows: exposed samples showed an inhibition of cellular iron metabolism, which caused p53 protein stabilization, which induced an increase of the transcription of miR-34a, decreasing the expression of E2F1/E2F3, thus affecting cell proliferation and cell senescence. It should be interesting in the future to evaluate, using the same experimental conditions, the effects of the exposure on other cancer cells.

### Investigations on brain cells

Several studies have suggested that ELF-MF exposure can be associated with an increased risk of neurodegenerative diseases, mainly Alzheimer’s disease (AD), amyotrophic lateral sclerosis (ALS) (Qiu et al. 2004; Consales et al. 2012; Jalilian et al. 2018; Huss et al. 2018) and Parkinson’s disease (PD) (Brouwer et al. 2015; Pedersen et al. 2017). On the other side, some evidence has shown that ELF-MF exposure can modulate endogenous neurogenesis, suggesting that it could be taken in consideration to treat neurological disorders (Piacentini et al. 2008; Cuccurazzu et al. 2010; Podda et al. 2014).

Four studies are reported below, whose research question was to explore if ELF-MF exposure could cause epigenetic alterations inducing effects on brain cells. To this aim, epigenetic marks in ELF-MF-exposed cells were analysed in vitro (Leone et al. 2014; Consales et al. 2018; Benassi et al. 2019) or in vivo (Erdal et al. 2018).

Leone et al. (2014) exposed mouse hippocampal neural stem cells (NSCs) to ELF-MF (50 Hz, 1 mT) and after exposure analysed numerous endpoints including histone modifications. They observed that ELF-MF exposure caused an enhancement of neuronal differentiation of NSCs and found that changes in histone acetylation were involved: in particular, ELF-MF-exposed samples showed an increased histone H3 acetylation at lysine 9 (H3K9) on the regulatory sequence of several pro-neuronal genes. Therefore, epigenetic modifications, particularly chromatin modifications at specific neuronal gene regulatory sequences, may be involved in the observed enhancement of hippocampal neurogenesis.

Consales et al. (2018) focused on the expression levels of three miRs (miR-133b; miR-34b and miR-34c arising from the common pri-miR-34b/c transcript), whose dysregulation has been associated with neurodegenerative diseases (Dardiotis et al. 2018). They used human neuroblastoma SH-SY5Y cells and mouse primary cortical neurons (PCNs), obtained from cerebral cortices of mice embryos. Cells were exposed to ELF-MF (50 Hz, 1 mT). The obtained results showed that the expression levels of miR-34b, miR-34c and pri-miR-34b/c decreased in ELF-MF-exposed cells compared to sham controls and that this decrease occurred starting from 24 h of exposure and maintained up to 72 h; differently, no change in miR-133b level was detected. In a more recent paper (Consales et al. 2021), also the expression levels of miR-21-5p and miR-222-3p were analysed and found unchanged. To investigate the potential mechanism of the deregulation of miR-34b, miR-34c and pri-miR-34b/c, the DNA methylation level of the CpG island of the pri-miR-34b/c promoter was evaluated. ELF-MF-exposed samples showed a significantly increase of methylation levels. Incubation with 5-aza-2deoxycytidine, a DNMT inhibitor, efficiently reverted ELF-MF-induced pri-miR-34 silencing. Therefore, the authors concluded that the reduction of the expression of both miR-34b and miR-34c was caused by a decreased transcription of the common pri-miR-34 due to the hypermethylation of the promoter. The in silico prediction of putative miR-34b/c targets, carried out by using miR databases and prediction algorithms, highlighted a consistent set of transcripts, including α-synuclein (Snca/Park1), a protein implicated in the aetiopathogenesis of PD (Bendor et al. 2013). The 3′UTR region of human Snca mRNA contains miR-34b/c binding sites (Kabaria et al. 2015). In the experiments performed by Consales et al. (2018), both Snca mRNA and protein levels increased upon ELF-MF exposure. Coherently, Snca transcript and protein levels were reduced in ELF-MF-exposed cells with the addition of an miR-34b mimic and increased in sham cells with the addition of anti-miR-34b. However, ELF-MF exposure induced Snca expression also in mouse PCNs, even though the 3′UTR of mouse Snca mRNA does not contain any miR-34b/c putative binding site; therefore, it was deduced that some other mechanisms, in addition to miR regulation, probably affect Snca expression.

The same research group in a recent study (Benassi et al. 2019) investigated whether exposure to ELF-MFs (50 Hz, 1 mT) could affect the global DNA methylation of SH-SY5Y cells under either basal culture conditions (proliferating) or differentiating treatment (retinoic acid, RA and phorbol 12-myristate 13-acetate (PMA)). Cells were cultured either in the presence or in the absence of neurotoxic stress induced by 1-methyl-4-phenylpyridinium (MPP^+^), a neurotoxin mimicking the PD phenotype (Benassi et al. 2016). MPP^+^ was administered 24 h after exposure to ELF-MFs and left in the medium for another 24 h until analyses. After ELF-MF exposure and control/neurotoxic treatment, the DNA methylation percentage for repetitive elements, including LINE-1, SATα and ALU, was measured. No significant difference was found between ELF-MF-exposed and exposed samples for all the experimental conditions tested. The authors concluded that the exposure to 50 Hz MF does not affect global DNA methylation in proliferating and dopaminergic differentiated SH-SY5Y cells, either under basal culture conditions or under neurotoxic stress.

In vivo study was carried out by Erdal et al. (2018), who investigated whether ELF-MF could affect the expression levels of the miRs that are associated with several brain disorders such as AD and schizophrenia (Hauberg et al. 2016; Kumar and Reddy 2016). To this purpose, male/female young and mature rats were exposed to ELF-MF (50 Hz, 1 mT); after 60-day exposure, the levels of six miRs (miR-9-5p, miR-26b-5p, miR29a-3p, miR-106b-5p, miR-107 and miR-125a-3p) were analysed in rat brain and blood samples. The animals used were male and female, young and mature, since previous studies reported that miR expression levels could change with age and sex (Morgan and Bale 2012). Results indicated that all the six miRs were differentially expressed in response to ELF-MF depending on cell type (brain or blood), age and sex (Table 2). The young female rats exposed to ELF-MF showed a significant decrease of the most part of the miRs analysed, whereas no statistically significant difference was observed in the mature females. Young and mature male rats showed some significant changes of miR levels both in the blood and in the brain tissue. Results of this study confirm that effects depend strongly on the cell type. The authors concluded that results provide evidence that long-time MF exposure in young rat, mainly female, can influence the expression levels of miRs in blood and brain and may be a risk factor for some neurological disease. However, it is important to underline that miR regulation can be different between humans and rats.

The four papers reported below (Pasi et al. 2016; Capelli et al. 2017; Giorgi et al. 2017; Yao et al. 2019) used pulsed electromagnetic fields (PEMFs), which are characterized by a rapid rise of the magnetic field, resulting in higher induced currents. PEMFs are considered potentially useful for therapeutic purposes and have been also used in clinical applications because of their efficacy (Arendash et al. 2010; Di Lazzaro et al. 2013; Xu et al. 2016; Ehnert et al. 2018).

Giorgi et al. (2017) analysed the DNA methylation levels of the 5′UTR of LINE-1 of human neural cells (BE(2)C) exposed to PEMF (50 Hz, 1 mT) in the presence or in the absence of oxidative stress (OS). The comparison of methylation levels of 24 CpGs among the samples showed that the methylation level of several CpG units was modified depending on the type of treatment: exposure to PEMF or to OS-induced weak alterations of DNA methylation levels at three different CpGs; differently, the combined exposure to PEMF and OS caused a significant decrease of DNA methylation levels at five different CpG sites. However, most of the alterations were transient, indicating that cells can restore homeostatic DNA methylation patterns.

The other three studies aimed at verifying if PEMF exposure could modulate the expression of miRs known to be involved in brain cancer (Pasi et al. 2016), in AD (Capelli et al. 2017) and in demyelinating disorder (Yao et al. 2019), in order to find a therapeutic tool for these diseases.

Pasi et al. (2016) focused on miR-17-3p, miR-21-3p and miR-421-5p, which are respectively related to antioxidant, cell cycle progression and DNA damage repair. All these miRs have been reported to be upregulated in several human cancer types. T98G glioblastoma cells, which are resistant to chemo- and radio-therapy, were exposed to PEMF (75 Hz, 2 mT) for 1 h and then subjected or not to treatment with temozolomide (TMZ), a chemotherapy drug used to treat some brain tumours. The expression of these miRs was studied 48 h after the end of TMZ/PEMF treatment. Changes of the expression levels were observed (Table 2): the most relevant result was that the expression of miRs was drastically reduced when the cells were treated with TMZ immediately after PEMF exposure. This observation indicates that PEMF coupled with chemotherapy (TMZ plus PEMF) can trigger epigenetic mechanisms to slow down the neoplastic proliferation.

Capelli et al. (2017) focused on miR-335-5p and miR-26b-5p, which are upregulated in AD disorder (Bottero and Potashkin 2019). They applied an ex vivo model: peripheral blood mononuclear cells (PBMCs) freshly isolated from the peripheral blood of AD patients were exposed to PEMF (75 Hz, 3 mT) and, after exposure, miR levels were evaluated. A progressive reduction of the two miRs with the increasing time of exposure was observed, even if the differences between untreated and treated cells were not statistically significant. These preliminary data suggest that ELF-MF exposure could normalize the expression of those miRs which are typically dysregulated in AD. Further research is needed, increasing the number of patients, to evaluate the significance of the data.

Yao et al. (2019) focused on miR-219-5p expression, which is associated with oligodendrocyte precursor cell (OPC) differentiation. After exposing primary rat OPCs to PEMF (50 Hz, 1.8 mT), they evaluated cell differentiation, through stage-specific marker analysis, and miR expression. Exposed samples exhibited accelerated OPC differentiation and higher relative expression of miR-219-5p than the control ones, indicating that PEMF exposure promoted the differentiation of OPCs via miR-219-5p upregulation. Thus, it was suggested that PEMF stimulation might have a potentially positive impact on the functional recovery process following severe traumatic demyelinating disorder.

### Investigations on induced pluripotent stem cell generation

It is well known that somatic cells can be reprogrammed to induced pluripotent stem cell (iPSCs) by the overexpression of the four “Yamanaka factors” (octamer-binding transcription factor 4, Oct4; sex determining region Y-box 2, Sox2; Kruppel-like factor 4, Klf4; and c-Myc) (Takahashi et al. 2007).

Baek et al. (2014) reported that mouse fibroblasts, when exposed to ELF-MF (50 Hz, 1 mT), could be reprogrammed to iPSCs, in the presence of the expression of only one of the Yamanaka factors (Oct4). The authors found that ELF-MF caused upregulation of the KMT2D gene that encodes the lysine-specific methyltransferase myeloid/mixed-lineage leukaemia 2 (Mll2). Mll2 is a member of the trithorax (trxG) group, responsible for histone modifications during development. Mll2 overexpression led to a significant induction in H3K4me3 levels, which increased accessibility of several pluripotency-associated loci. Therefore, Mll2 could be a key mediator of the effects of electromagnetic fields during reprogramming.

Interestingly, the same experiment was repeated by using a magnetic field–free system and it was found that generation of iPSC colonies was delayed through the suppression of epigenetic reprogramming. This finding indicates that the environmental electromagnetic field energy is essential for favourable epigenetic remodelling during the acquisition of pluripotency. Later, the same research group (Baek et al. 2019) studied the differentiation of mouse embryonic stem cells (ESCs) under hypomagnetic field (HMF) conditions and found that HMF delayed cell fate conversion during ESC differentiation through genomic DNA methylation pattern alteration: this result is a further confirmation of the fact that electromagnetic fields play a role in epigenetic regulation.

### Investigations on osteogenic differentiation

It is widely accepted that PEMF exposure can promote osteogenesis, stimulating the osteoblast differentiation of human bone mesenchymal stem cells (hBMSCs). Indeed, this exposure has been successfully applied to improve bone regeneration in skeletal diseases and fractures (Ongaro et al. 2014; Varani et al. 2021). In order to investigate the molecular mechanisms behind this phenomenon, De Mattei et al. (2020) assessed whether PEMF exposure can modulate the expression of miRs involved in osteogenic differentiation. hBMSCs were cultured in an appropriate osteogenic differentiation medium and then exposed to PEMF (75 Hz, 1.5 mT) for 21 days. At the end of the experiment, it was found that PEMFs regulated three miRs (miR-26a, miR-29b, miR-25b) that are involved in different phases of osteogenic differentiation and bone repair.

#### Conclusions

We found only 15 experimental studies that evaluate the effects of ELF-MF exposure on epigenetic marks. These studies are very heterogeneous in duration (from 1 h to 60 days), mode of the exposure (continuous or intermittent) and physical characteristics of ELF-MF. Indeed, the magnetic field direction (changing continuously in RMF with respect to sinusoidal and pulsed fields), its rise (rapid in PEMF and smooth in sinusoidal alternating fields), the frequency itself and the intensity values are all parameters that might lead to different effects (IARC 2002).

Moreover, it is worth noting that some experimental conditions simulate the exposure which we are subjected to in our daily lives, whereas other types (PEMF, RMF) hardly occur in a normal environment but are investigated for therapeutic purposes.

The central role of the earth magnetic field in epigenetic reprogramming, found by Baek et al. (2014, 2019), suggests that any exposure to man-made electromagnetic fields (which may be thousands of times greater than the natural one) might be critical for the epigenetic changes.

The molecular mechanisms through which the various types of electromagnetic fields interact with organic molecules are not yet clear. One hypothesis is that the field induces changes in the energy levels of certain molecules through the radical-pair mechanism (IARC 2002; Barnes and Greenebaum 2015; Sherrard et al. 2018). This may affect concentration of free radicals, such as reactive oxygen species (ROS). Changes in oxidative status have been observed in a broad range of cell types subjected to various exposure types (Mattsson and Simkó 2014). ROS can modulate cell signalling (Finkel, 2011), leading to biologically significant changes, including epigenetic ones (Afanas'ev, 2014). Therefore, ROS could be involved in ELF-MF-induced epigenetic changes (Consales et al. 2018; Merla et al. 2019). ELF-MFs might interact with membrane targets, such as transmembrane ion channels, including those involved in calcium metabolism regulation (Golbach et al. 2016). Calcium signalling plays a role in gene expression and is also important in epigenetic regulation (Puri 2020). However, for the time being, the chain of molecular events leading to epigenetic dysregulation is still unknown.

Although data collected in the present review are still too few and varied to draw any conclusion, they are worth a closer examination.

Most of the studies (13 out of 15) observed that ELF-MF exposure can induce an alteration of epigenetic marks. When different researcher groups assessed the same marks (i.e. miR-26b-5p, miR-29b, miR-107, miR-335), all of them found alterations in the exposed samples, although different kind of changes (increase or decrease) were observed with different models and different exposure conditions.

Of great interest are the results reported by four papers, dealing with the effects of ELF-MF during cell differentiation (Leone et al. 2014; Yao et al. 2019; De Mattei et al. 2020) or iPSC generation (Baek et al. 2014). They found that the exposure respectively promoted cell differentiation and iPSC generation. It was already known that electromagnetic fields can contribute to reprogramming of human skin fibroblasts (Ventura et al. 2005; Maioli et al. 2013) and can affect biological processes such as embryogenesis, regeneration and cell fate conversion (Maziarz et al. 2016): the novelty of the above four studies is the finding that ELF-MFs affect these processes through epigenetic alterations. Indeed, differentiating and dedifferentiating cells are subjected to global changes in the epigenetic landscape; for this reason, they are highly sensitive to alterations induced by environmental agents (including ELF-MF) as compared to somatic differentiated cells, which are generally more epigenetically stable.

Some effects have been observed also in differentiated cells, but it is unclear whether these effects are transient or not and which are the potential long-term consequences for cell biological functionality.

For the most part, data summarized here were obtained using in vitro systems consisting of monolayer cultures of cell lines, which are neoplastic cells. These models show some limitations: cancer cells exhibit numerous anomalies; in addition, conventional monolayer cultures lack the complexity of in vivo conditions. There are a few promising studies, regarding the biological effects of ELF-MF exposure, that promote the use of 3D cell systems, thus providing more physiological conditions as compared to the conventional 2D cell cultures (Hilz et al. 2014; Bai et al. 2017; Consales et al. 2021). Future research directions should be oriented to the use of nonneoplastic cells in 3D cell cultures.

Overall, current results constitute a good basis for future investigations and suggest that ELF-MF exposure could induce epigenetic alterations with major effects on cells undergoing differentiation and dedifferentiation processes. Further research is needed to understand the underlying molecular mechanisms. The acquisition of more knowledge on this topic could provide a basis both to develop therapeutic strategies and to prevent health hazards.
